# Elastolysis in COPD: a Target for Therapy

**DOI:** 10.1007/s00408-024-00693-3

**Published:** 2024-04-27

**Authors:** Gerard M. Turino, Jerome O. Cantor

**Affiliations:** 1 https://ror.org/04a9tmd77 grid.59734.3c 0000 0001 0670 2351 Mount Sinai Icahn School of Medicine, New York, NY USA; 2 https://ror.org/00bgtad15 grid.264091.8 0000 0001 1954 7928 . John’s University, Queens, NY USA

The purpose of this perspective is to indicate the mounting evidence that elastin degradation of the lung is the primary etiological mechanism in the pathogenesis of Chronic Obstructive Pulmonary Disease (COPD) and should be a focus of diagnosis and therapy. The clinical characteristics of the disease include chronic bronchitis, airway obstruction, hyperinflation of the lungs and emphysema [1]. These pathophysiological alterations result in physical disability and early mortality [2]. Among the most prominent morphological changes are airway inflammation, fragmentation of elastin in bronchi and lung parenchyma and rupture of alveolar walls [3, 4].

Insights into the anatomical and chemical structure of elastin demonstrated that elastin is anchored in tissue by the unique desmosine and isodesmosine (DI) crosslinks that function and exist only in elastin [5]. Consequently, DI have become recognized as biomarkers for elastin degradation in body organs [6]. Studies of short term and long-term replacement therapy of Alpha-1 Antitrypsin protein in Alpha-1 Antitrypsin deficiency disease repeatedly show statistical reductions in DI levels [7–9]. Recently reported long-term DI biomarker studies indicate elevated levels of DI in urine in several hundred patients with COPD followed for many years [10, 11]. The significance of these large biomarker studies is the consistency of increased levels of the biomarker despite variations in the patterns of clinical and physiological characteristic of COPD in these large patients’ populations. These results justify a focus on elastin degradation as a primary pathological mechanism for diagnosis and a specific target for therapy.

Early methods of analysis and quantification of DI involved use of radioimmunoassay techniques which gave variable results [12]. The introduction of mass spectrometry and liquid chromatography to the analytical techniques has improved accuracy and sensitivity [13]. As a result, DI have received increasing application as a biomarker for COPD, resulting in two large studies over recent years [10, 11]. These biomarker results have been uniformly positive in relation to the death rate in COPD and the correlations with the degree of airway obstruction. The large COPD patient populations in biomarker studies include the very variable clinical and physiological characteristics of the disease in patients with COPD. Increased amounts of DI in sputum, plasma, and urine are consistently observed despite these differences in clinical characteristics. Increased levels of DI in body fluids have therefore evolved as a common denominator of COPD pathogenesis and should be recognized as such.

The variability of the clinical and physiological characteristic of patients with COPD can result from variations in pollutant exposure, living habits and innate (DNA) variability. In this regard emphasis in the published literature of the clinical variability of COPD does not provide insights into the basic mechanism of COPD which is elastin degradation in all parts of the lung and is the primary cause of emphysema. Further insights into the role of elastin degradation in the pathogenesis of pulmonary emphysema have come from animal studies which show the development of pulmonary emphysema from long-term tobacco smoke exposure resulting in lung elastin degradation and preservation of lung elastin by exposure to hyaluronan aerosol [14]. It is also noteworthy, that elastin peptides which result from elastin degradation contribute significantly to lung parenchymal inflammation as an ongoing mechanism for elastin degradation [15].

A major insight into the causes of emphysema was provided by the discovery of the genetic abnormality Alpha-1 Antitrypsin Deficiency (ATTD) in 1963 which causes pulmonary emphysema in early adulthood and is caused by a failure to inactivate neutrophil elastase [16].

Recent studies indicate statistically significant positive therapeutic results of replacement therapy in (ATTD) [17, 18].

These new insights into the role of lung elastin degradation deserve increased recognition in studies going forward. In this regard elastin degradation should become a target for therapy to prevent and control lung injury in COPD.

## Data Availability

No datasets were generated or analysed during the current study.
